# Small RNAs Derived from the T-DNA of *Agrobacterium rhizogenes* in Hairy Roots of *Phaseolus vulgaris*

**DOI:** 10.3389/fpls.2017.00096

**Published:** 2017-02-01

**Authors:** Pablo Peláez, Alejandrina Hernández-López, Georgina Estrada-Navarrete, Federico Sanchez

**Affiliations:** ^1^Departamento de Biología Molecular de Plantas, Instituto de Biotecnología, Universidad Nacional Autónoma de MéxicoCuernavaca, Mexico; ^2^Laboratorio Nacional de Genómica para la Biodiversidad, Unidad de Genómica Avanzada del Centro de Investigación y de Estudios Avanzados del Instituto Politécnico NacionalIrapuato, Mexico

**Keywords:** *Agrobacterium rhizogenes*, hairy roots, RNA silencing, small RNAs, T-DNA

## Abstract

*Agrobacterium rhizogenes* is a pathogenic bacteria that causes hairy root disease by transferring bacterial DNA into the plant genome. It is an essential tool for industry and research due to its capacity to produce genetically modified roots and whole organisms. Here, we identified and characterized small RNAs generated from the transfer DNA (T-DNA) of *A. rhizogenes* in hairy roots of common bean (*Phaseolus vulgaris*). Distinct abundant *A. rhizogenes* T-DNA-derived small RNAs (ArT-sRNAs) belonging to several oncogenes were detected in hairy roots using high-throughput sequencing. The most abundant and diverse species of ArT-sRNAs were those of 21- and 22-nucleotides in length. Many T-DNA encoded genes constituted phasiRNA producing loci (*PHAS* loci). Interestingly, degradome analysis revealed that ArT-sRNAs potentially target genes of *P. vulgaris*. In addition, we detected low levels of ArT-sRNAs in the *A. rhizogenes*-induced calli generated at the wound site before hairy root emergence. These results suggest that RNA silencing targets several genes from T-DNA of *A. rhizogenes* in hairy roots of common bean. Therefore, the role of RNA silencing observed in this study has implications in our understanding and usage of this unique plant-bacteria interaction.

## Introduction

*Agrobacterium rhizogenes* and *Agrobacterium tumefaciens* are plant pathogenic bacteria capable of inducing uncontrolled cell proliferation and development of hairy roots and crown gall tumors, respectively. During the infection process, a transferred DNA from the bacteria root inducing (Ri) or tumor-inducing (Ti) plasmids is integrated into the plant cells genome (Tzfira and Citovsky, 2006). The genes encoded in the T-DNA (oncogenes) lead to abnormal proliferation of cells through changes in signal transduction pathways and transcription factors that affect the production of plant hormones (Britton et al., 2008). Transformed cells produce amino acid and sugar derivatives (opines) that are used by bacteria as nutrients and trigger conjugal transfer of T-DNA-carrying plasmids (Subramoni et al., 2014). The unique properties of these bacteria to perform cross-kingdom DNA transfer have been widely exploited to transform plants. *A. tumefaciens* strains with Ti plasmids lacking T-DNA oncogenes do not induce tumor growth and have been used as efficient delivery systems for plant transformation (Krenek et al., 2015). Hairy roots harboring the *A. rhizogenes* Ri plasmid T-DNA have also become an important tool for basic research and biotechnology (Georgiev et al., 2012). Transgenic roots emerge from totipotent calli cells that, subsequently, differentiate into highly branched ageotropic roots. Whole plants regenerated from hairy roots exhibit a particular phenotype (called hairy root phenotype) that comprises loss of apical dominance, wrinkled leaves, reduced fertility, shortened internodes, stunted growth, and abnormal organs (Tepfer, 1984). Several studies have attempted to determine the precise function of the T-DNA genes from *A. rhizogenes* strains in the infection process and in the hairy root phenotype (Britton et al., 2008).

RNA silencing constitutes a defense mechanism against foreign genetic material, such as transgenes or viral nucleic acids (Hamilton and Baulcombe, 1999). Small RNAs of 21–24 nucleotides (nt) in length trigger post-transcriptional gene silencing (PTGS) or transcriptional gene silencing (TGS) of endogenous or foreign genetic elements (Baulcombe, 2004). RNA silencing is initially triggered by double-stranded RNA (dsRNA), which is cleaved by Dicer-like proteins (DCLs) to form small RNAs (sRNAs). These sRNAs are then loaded into Argonaute proteins (AGOs) to direct RNA or DNA silencing via base pairing complementarity (Chapman and Carrington, 2007). Furthermore, RNA-dependent RNA polymerases (RDRs) produce secondary sRNAs that supports systemic spread of RNA silencing through long distances within plants (Willmann et al., 2011).

Transgenes incorporated by *Agrobacterium*-mediated transformation are frequently subject to RNA silencing. Dunoyer et al. (2006) proposed that transgene silencing could be a defense response typically conducted against T-DNA oncogenes of virulent bacteria similar to that occurring during viral infections. Analyzing the defensive role of RNA silencing in virulent *A. tumefaciens* infections, they found that mutants of *dcl1* and plants deficient in miRNA functions became immune to *A. tumefaciens*. Accumulation and activity of microRNAs (miRNAs) in tumors were only moderately altered, highlighting the contribution of miRNA-regulated processes to tumor development. Besides, *Arabidopsis thaliana rdr6* mutants and viral suppressor expressing plants were more susceptible to the infection. Surprisingly, RNA silencing against T-DNA genes happens at the early stages of the infection but is inhibited in tumors. Small RNAs of 21-nt in length from the tryptophan 2-monooxygenase (*iaaM*) and the agropine synthase (*ags*) T-DNA genes were detected at 3 days post-infiltration of virulent *A. tumefaciens* in leaves of *Nicotiana benthamiana*; however, no sRNAs were detected in tumors. The authors proposed that suppression of RNA silencing pathways in *A. tumefaciens*-induced tumors could be an event intrinsic to dedifferentiation and/or proliferation probably caused by phytohormones (Dunoyer et al., 2006). In this regard, the 6b protein from the T-DNA region of the Ti plasmid was reported to interact with AGO1 and the SERRATE (SE) protein involved in the biogenesis of miRNAs (Wang et al., 2011). The reduced accumulation of miRNAs observed in plants overexpressing the 6b protein suggests that it could act as a suppressor of RNA silencing and may contribute to the RNA silencing suppression state observed in tumors (Wang et al., 2011). Interestingly, the 6b protein is not present on the T-DNA of the Ri plasmid of *A. rhizogenes* (Britton et al., 2008). Recently, small RNAs from the T-DNA of *A. rhizogenes* were detected in *Nicotiana tabacum* plants that acquired the T-DNA naturally through horizontal gene transfer (Kovacova et al., 2014).

Hairy roots are differentiated roots that have been proven to be a powerful tool for loss-of-function analyses of genes in many species via RNA silencing. They have been generally employed in plants recalcitrant to *A. tumefaciens*-mediated transformation, such as common bean. Nevertheless, *A. rhizogenes*-induced hairy roots should not have the RNA silencing pathways suppressed like *A. tumefaciens*-induced tumors. Genes from the T-DNA of *A. rhizogenes* could be regulated by the RNA silencing mechanism in this biological system and small RNAs belonging to these genes could be detected. Herein we report *A. rhizogenes* T-DNA-derived small RNAs (ArT-sRNAs) in hairy roots of common bean. We characterized these T-DNA derived small RNAs and discussed their relevance to plant-bacteria interactions. Distinct abundant *A. rhizogenes* T-DNA-derived small RNAs belonging to several oncogenes and to the opine synthase gene were detected. Our data demonstrates that many T-DNA encoded genes constitute phasiRNA-producing loci probably subjected to PTGS triggered by dsRNA-derived ArT-sRNAs of 21- and 22-nt in length. We also found that these sRNAs generated from bacteria's genetic material may target host genes. Our results indicate that RNA silencing may regulate T-DNA genes of the Ri plasmid of *A. rhizogenes* in hairy roots of common bean, suggesting that RNA silencing may play an important role in this naturally occurring plant transformation.

## Materials and methods

### Plant material

Hairy roots and calli were obtained from *Phaseolus vulgaris* L. cv. Negro Jamapa adjusting the procedure described by Estrada-Navarrete et al. (2007). Ten days after infection with *A. rhizogenes* K599, the primary root was removed. Then plants were grown in hydroponics (B&D solution supplemented with 8 mM KNO_3_, pH 6.5) for 11 days in a chamber with 16 h of light and 8 h of dark at 28°C until hairy roots collection. This differs from Estrada-Navarrete et al. (2007) protocol (step 20). For 6 days old calli induction and collection, seeds were germinated as Estrada-Navarrete et al. (2007) protocol (steps 1–7). Then, after seedlings were grown in hydroponics for 2 days with B&D solution, bacteria (steps 9–10) was inoculated in the stem (not in the cotyledonary nodes). Seedlings were grown 6 days in hydroponics inside a chamber to preserve humidity. Common bean non-transgenic roots were obtained from germinated seeds (steps 1–7) grown for 4 days in hydroponics. All samples were frozen in liquid nitrogen and stored at −80°C until RNA extraction.

### RNA extraction and library sequencing

Total RNA was extracted from hairy roots and calli using the Trizol reagent (Invitrogen). Ten micrograms of each sample were prepared for deep sequencing of small RNA libraries. Sequences ranging from 18 to 30 nt were purified for the construction of the libraries. A small RNA library of hairy roots was prepared following Illumina's Small RNA alternative sample preparation protocol v1.5. The small RNA library of calli was constructed according to the Illumina's TruSeq Small RNA Sample Prep Kit. Libraries were Single Read-sequenced using the Genome Analyzer IIx (GAIIx) and the Illumina Cluster Station at the Instituto de Biotecnología (Universidad Nacional Autónoma de México). Degradome library construction for the hairy roots sample was performed as described by Ma et al. (2010) with some changes. Approximately 150 ng of poly(A)^+^ RNA was used to anneal with biotinylated random primers. Strapavidin capture of RNA fragments was performed using biotinylated random primers. A 5′ adaptor ligation was only performed to those RNAs containing 5′-monophosphates. The library was sequenced with the Illumina's Cluster Station and the GAIIx using the 5′ adapter only. This resulted in the sequencing of the first 36 nt of the inserts that represent the 5′ ends of the original RNAs (LC Sciences). All sequence data is available at the European Nucleotide Archive (EMBL-EBI) with the accession number PRJEB7993.

### Bioinformatics and data analysis

Sequence length distribution and alignment (PatMaN; Prüfer et al., 2008) of small RNAs were performed using the UEA small RNA analysis toolkit (Version 2.5.0; Stocks et al., 2012). Small RNAs (18–26 nt) were aligned to the *A. rhizogenes* plasmid Ri T-DNA region without mismatches (GenBank accession number: EF433766). Whole T-DNA alignments were seen with the Integrative Genomics Viewer (IGV; Thorvaldsdóttir et al., 2013). RNA secondary structures of the T-DNA loci were predicted with the Vienna RNA secondary structure server using the minimum free energy algorithm (MFE; Hofacker, 2003). *P. vulgaris* genes targeted by T-DNA derived sRNAs were predicted *in silico* using the plant small RNA target analysis server psRNATarget (Dai and Zhao, 2011) with a maximum expectation threshold of 0–2. *P. vulgaris* (Phytozome v9.0) spliced mRNA transcripts without alternative splice variants were used for target prediction analysis. Multiple alignment of *rolA* sequences from different Ri plasmids (pRi1724/gb: AP002086, pRi8196/gb:M60490, pRiA4/gb:X12579) was performed using ClustalW2 with default settings (Larkin et al., 2007) and viewed with Jalview (Waterhouse et al., 2009). Previously reported microRNA sequences from *P. vulgaris* were used for the identification of microRNAs in the small RNA libraries and for the non-transgenic root miRNA accumulation analysis (Peláez et al., 2012). Normalized read frequencies (RPM) of miRNAs from the same family were added for miRNA accumulation analysis.

### Analysis of phasiRNAs

Two strategies were used for the identification of loci producing phasiRNAs of 21 nt in length (Chen et al., 2007; Axtell, 2010). The algorithm developed by Chen et al. (2007) was used through the UEA small RNA analysis toolkit implementation (**Figures 5B,C**). Only sRNAs derived from the T-DNA and the plasmid Ri T-DNA sequence (gb: EF433766) were used as input. Moreover, this algorithm was performed for the identification of phasing-generating loci from *P. vulgaris* used as controls (Supplementary Table 9). Combined abundances of two and five bins were used in the methodology developed by Axtell (2010) to calculate the phase ratio of *rolB* and *CUS* loci, respectively.

### Northern blot analysis

Total RNA was extracted using Trizol reagent (Invitrogen). RNA (20 ug) was separated by 15% PAGE/8 M urea/ 1x TBE buffer. Gels were electro-blotted to a Hybond-N^+^ membrane (GE Healthcare) and UV cross-linked twice. Oligonucleotide probes (Supplementary Table 10) were labeled using [γ-^32^P]-ATP (Perkin-Elmer) and T4 PNK (New England Biolabs). Labeled probes were purified with the Quick spin-oligo columns (Roche) before hybridization. Hybridizations were performed at 42°C overnight in UltraHyb-oligo solution (Ambion). After hybridization, membranes were washed twice in 2 × SSC/0.1% SDS and exposed to the Phosphor Screen System (GE Healthcare). The screen was scanned in a Storm 860 Gel and Blot Imaging System (GE Healthcare). As loading control, an oligonucleotide probe complementary to the U6 small nuclear RNA was used. Signal intensities were quantified using the ImageQuant 5.2 software (Molecular Dynamics). The U6 signal intensity in each blot was used for normalization and calculation of expression ratios. The same loading control was employed for membranes used more than once.

### Degradome data analysis

Perfect alignments of degradome sequences to the T-DNA were performed using PatMaN (Prüfer et al., 2008). The PAREsnip tool was used for the discovery of small RNA-guided cleavage sites with default parameters (Folkes et al., 2012). The small RNAs derived from the T-DNA, the T-DNA sequence (gb: EF433766) and the degradome sequences were used as input for the characterization of sRNA-mediated cleavages on the *A. rhizogenes* plasmid Ri T-DNA region. Primary transcripts (Phytozome v9.0) were used as input for the discovery of *P. vulgaris* targets cleaved by T-DNA derived sRNAs. Degradome sequences from seedlings of common bean were explored in the same way as input and used as control (Formey et al., 2015). Host targets were discarded if other sRNA from the library was also predicted to cleave the transcript and presented a better hit in at least one of the parameters evaluated such as alignment score, category, reads abundance, or *p*-value. *Arabidopsis* homolog targets were functionally grouped according to the Gene Ontology (GO) categories (Berardini et al., 2004). GO enrichment analysis was performed through the Plant GeneSet Enrichment Analysis Tool kit under default parameters (Yi et al., 2013). MicroRNAs (Supplementary Table 4) were used for analysis of validated target-sRNA interactions in the degradome library (Supplementary Table 5).

### Quantitative PCR

Detection and quantification of transcripts accumulation in hairy roots was performed using the iQ5 Real-Time PCR Detection System and the iQ5 Optical System Software (Bio-Rad). First strand cDNA was synthesized from DNA-free RNA with the RevertAid H Minus First Strand cDNA Synthesis kit (Fermentas). The qRT-PCR reactions were carried out in triplicate for three biological replicates with the Maxima SYBR green-fluorescein qPCR master mix (Fermentas). The gene-specific oligonucleotides belonging to the T-DNA genes were tested also on non-transformed tissues as controls (Supplementary Table 11). The melting temperature was set at 60°C. Quantification was based on a cycle threshold (Ct) value and the genes expression levels were normalized with the elongation factor 1 (*EF1*) reference gene.

## Results

### Small RNAs in the hairy root disease

**Figure 1:**
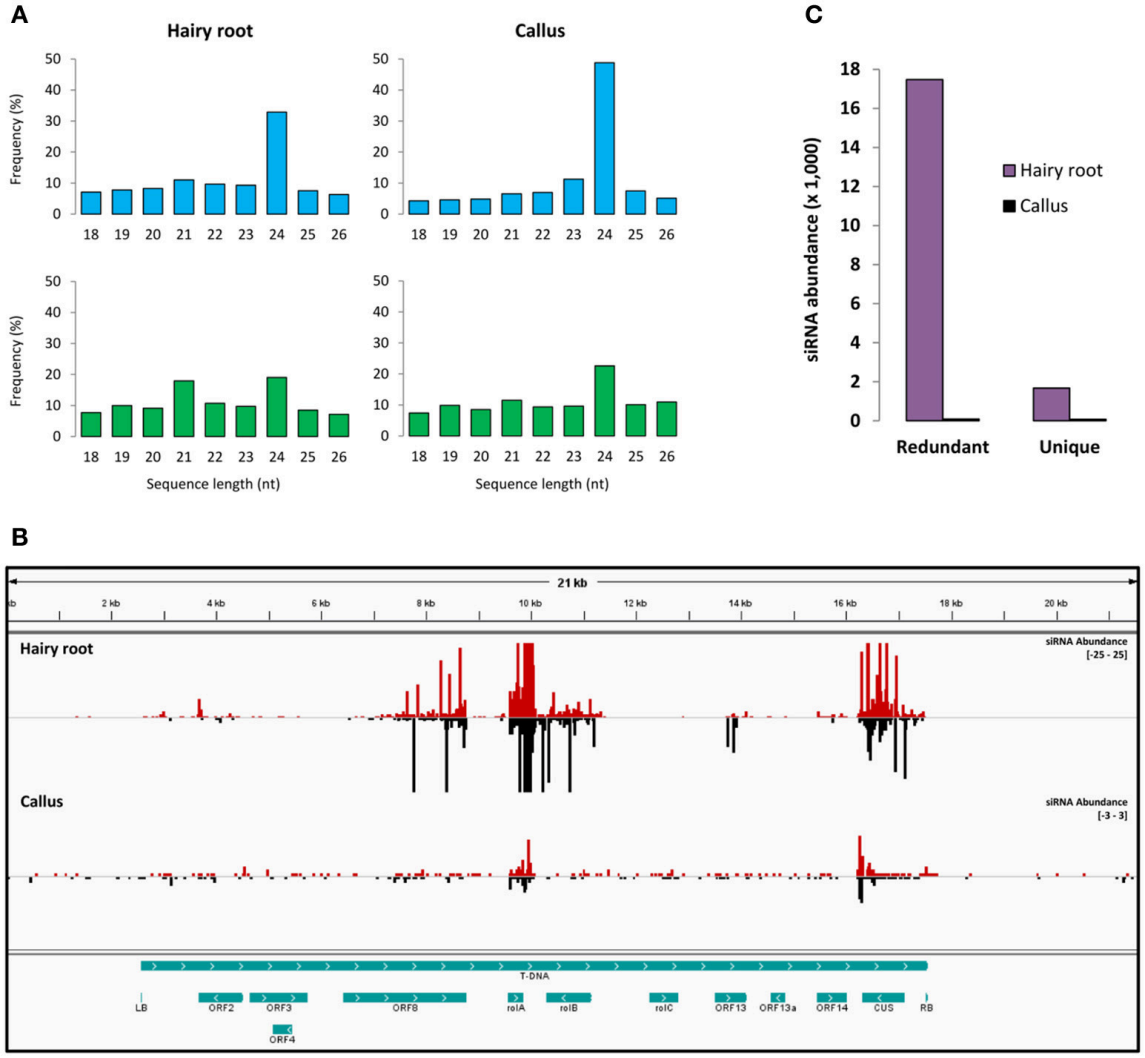
**Small RNAs in hairy roots and ***A. rhizogenes***-induced calli. (A)** Sequence length distribution of small RNAs. Average percentage (Y-axis) of unique (blue bars) and redundant (green bars) sequences of 18–26 nt length (X-axis) for the hairy roots and the *A. rhizogenes*-induced calli libraries. **(B)** Abundance and distribution of ArT-sRNAs in the T-DNA. The small RNAs that aligned to the plus strand (red bars) and to the minus strand (black bars) of the T-DNA of *A. rhizogenes* (greenish blue long bar) are indicated for the hairy roots (upper part) and calli (lower part) libraries. The abundance scale (right) differs between the libraries to show ArT-sRNAs in calli. In the bottom, the coding regions and the borders from the T-DNA are represented (greenish blue bars). **(C)** Comparison of the number of ArT-sRNAs detected in hairy roots and calli. Abundance levels were graphed as small RNA reads per million.

In order to identify small RNAs derived from the T-DNA of *A. rhizogene*s in the hairy root disease, small RNA libraries from hairy roots, and calli (formed before the emergence of hairy roots at the wound site) were generated. The presence and expression of several genes from the T-DNA in hairy roots (Supplementary Figure 1) were confirmed. Small RNA sequencing yielded 12,396,242 and 27,272,479 total raw reads for the hairy roots and calli samples, respectively. Small RNA sequences (18–26 nt) in hairy roots showed that the 21-nt (11%) and the 24-nt (32%) classes were the most abundant (redundant sequences). The 21-nt class was the second most diverse (unique sequences) in hairy roots and the fourth in *A. rhizogenes*-induced calli (Figure 1A). Perfect sequence alignment of small RNAs against the T-DNA of *A. rhizogenes* revealed the presence of several distinct and abundant ArT-sRNAs (18–26 nt) in hairy roots (Figure 1B). In hairy roots, 3176 distinct ArT-sRNAs corresponding to 17,000 small RNA reads per million (RPM) were detected. In contrast, only 563 ArT-sRNAs (unique sequences) corresponding to an abundance of 90 RPM were derived from T-DNA in calli (Figure 1C; Supplementary Table 1; De Paoli et al., 2009). Differences in the amount of ArT-sRNAs detected between hairy roots and calli may be a consequence of the amount of transformed cells in each sample. Most of the ArT-sRNAs observed in hairy roots aligned with transcriptional units of the T-DNA belonging to genes, such as *ORF2, ORF8, rolA, rolB, ORF13, ORF14*, and the opine synthase gene *CUS* (Figure 1B; Table 1), with the oncogene *rolA* being the major source of ArT-sRNAs in hairy roots. Interestingly, virtually no ArT-sRNAs derived from the oncogene *rolC* were detected in hairy roots. Also, nothing but 134 unique ArT-sRNAs were shared between hairy roots and calli.

**Table 1:** **Number and location of raw small RNA reads derived from the T-DNA**.

**Region (T-DNA)**	**Gene(s)**	**Hairy roots**		**Callus**	
		**Unique**	**Redundant**	**Unique**	**Redundant**
2596–3684		44	66	20	23
3685–4509	ORF2	57	71	12	13
4510–4652		0	0	6	8
4653–5747	ORF3 and ORF4	13	13	11	12
5748–6434		2	2	5	5
6435–8777	ORF8	485	1,334	60	65
8778–9577		23	30	6	6
9578–9859	rolA	351	1,015	83	104
9860–10307		924	26,935	59	89
10308–11147	rolB	314	774	18	19
11148–12272		24	56	15	16
12273–12815	rolC	0	0	19	20
12816–13510		1	1	10	10
13511–14107	ORF13	40	93	17	17
14108–14571		4	4	5	5
14572–14844	ORF13a	2	2	3	3
14845–15453		1	1	7	7
15454–16020	ORF14	80	90	8	8
16021–16331		75	175	39	82
16330–17121	CUS	623	2,165	110	125
17122–17527		113	162	17	19

*Unique and redundant raw reads from hairy roots and A. rhizogenes-induced calli small RNA libraries generated from coding and non-coding regions of the T-DNA of A. rhizogenes (region according to base-pair positions in gb: EF433766)*.

**Figure 2:**
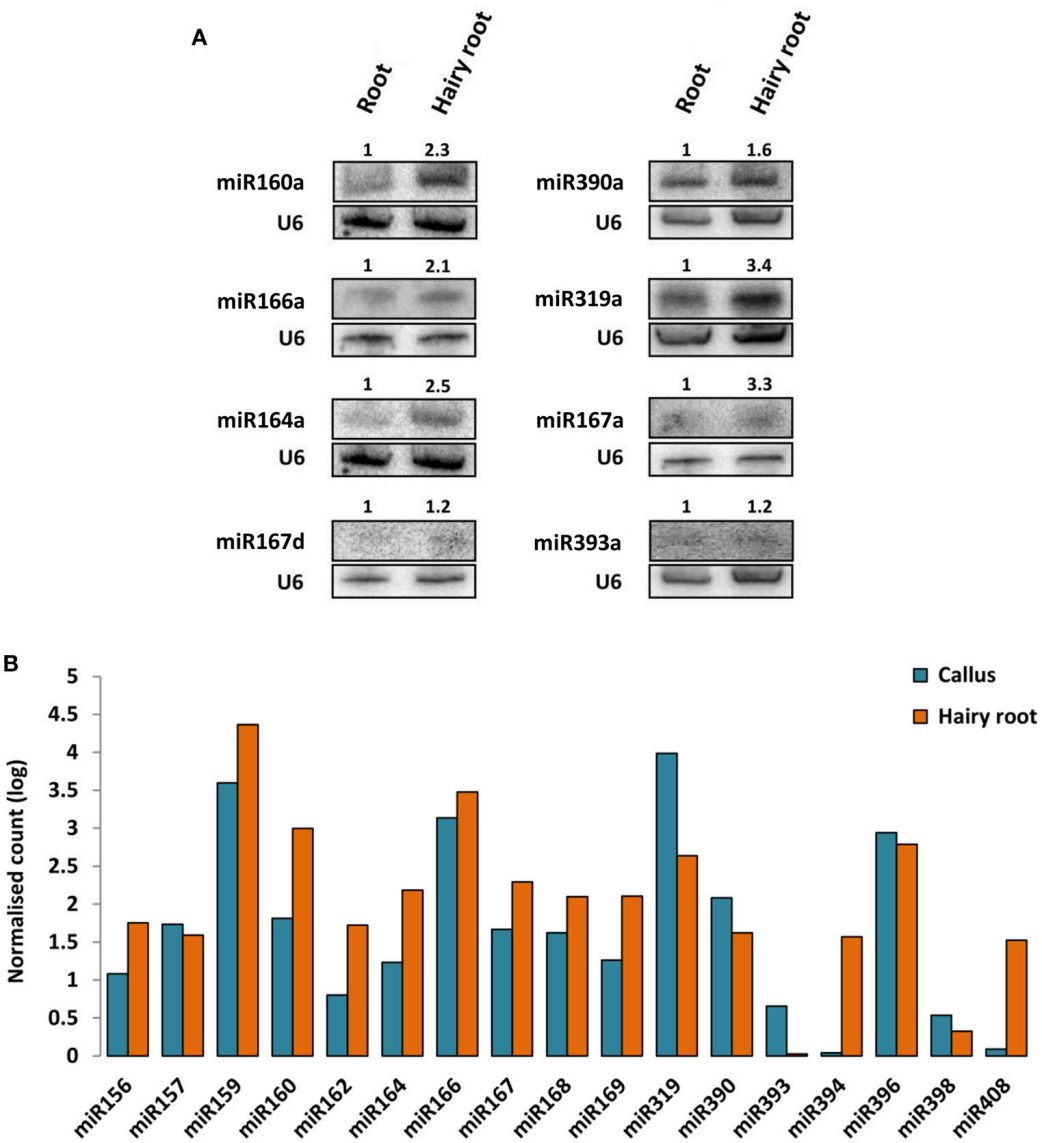
**Accumulation of microRNAs in non-transgenic root, hairy roots and ***A. rhizogenes***-induced calli. (A)** Northern blot analysis of microRNAs in hairy roots and non-transgenic roots of common bean. The signal of the U6 small nuclear RNA was used as loading control. The accumulation ratios between non-transgenic roots and hairy roots were calculated (upper numbers in gels). **(B)** Differential accumulation analysis of microRNAs from hairy roots and calli libraries. Frequencies of reads were normalized (RPM) and represented in logarithmic scale (Y-axis).

Moreover, the impact of ArT-sRNAs production on the accumulation of microRNAs in hairy roots and calli was measured. Members of conserved miRNA families, such as miR156, miR157, miR159, miR160, miR162, miR164, miR166, miR167, miR168, miR169, miR319, miR390, miR393, miR394, miR396, and miR408 were considered. Production of miRNAs in hairy roots was similar compared with non-transgenic roots according to northern blot analysis for eight miRNAs (Figure 2A). Relative miRNA expression estimation using the frequencies of miRNAs detected in the hairy roots and the calli libraries showed that the majority of miRNAs analyzed accumulated less in calli than in hairy roots. Substantial differences in accumulation were detected in these two samples for microRNAs miR394, miR408, and miR319 (Figure 2B). These differences in accumulation were also observed for these three families of microRNAs between the read frequencies of calli and a previously reported small RNA library from non-transgenic roots (Supplementary Figure 2; Peláez et al., 2012). MicroRNAs miR408 and miR394 were up-regulated in hairy roots and non-transgenic roots compared with calli, whereas miR319 was down-regulated in both type of roots. Also, miR393, a microRNA involved in plant defenses and auxin signaling, was more abundant in calli than in hairy roots (Figure 2B; Peláez and Sanchez, 2013). Interestingly, miR319 was the most abundant miRNA detected in calli. This was not the case for small RNA libraries of common bean from roots, leaves, flowers, and seedlings (Peláez et al., 2012).

### *Agrobacterium rhizogenes* T-DNA-derived small RNAs

**Figure 3:**
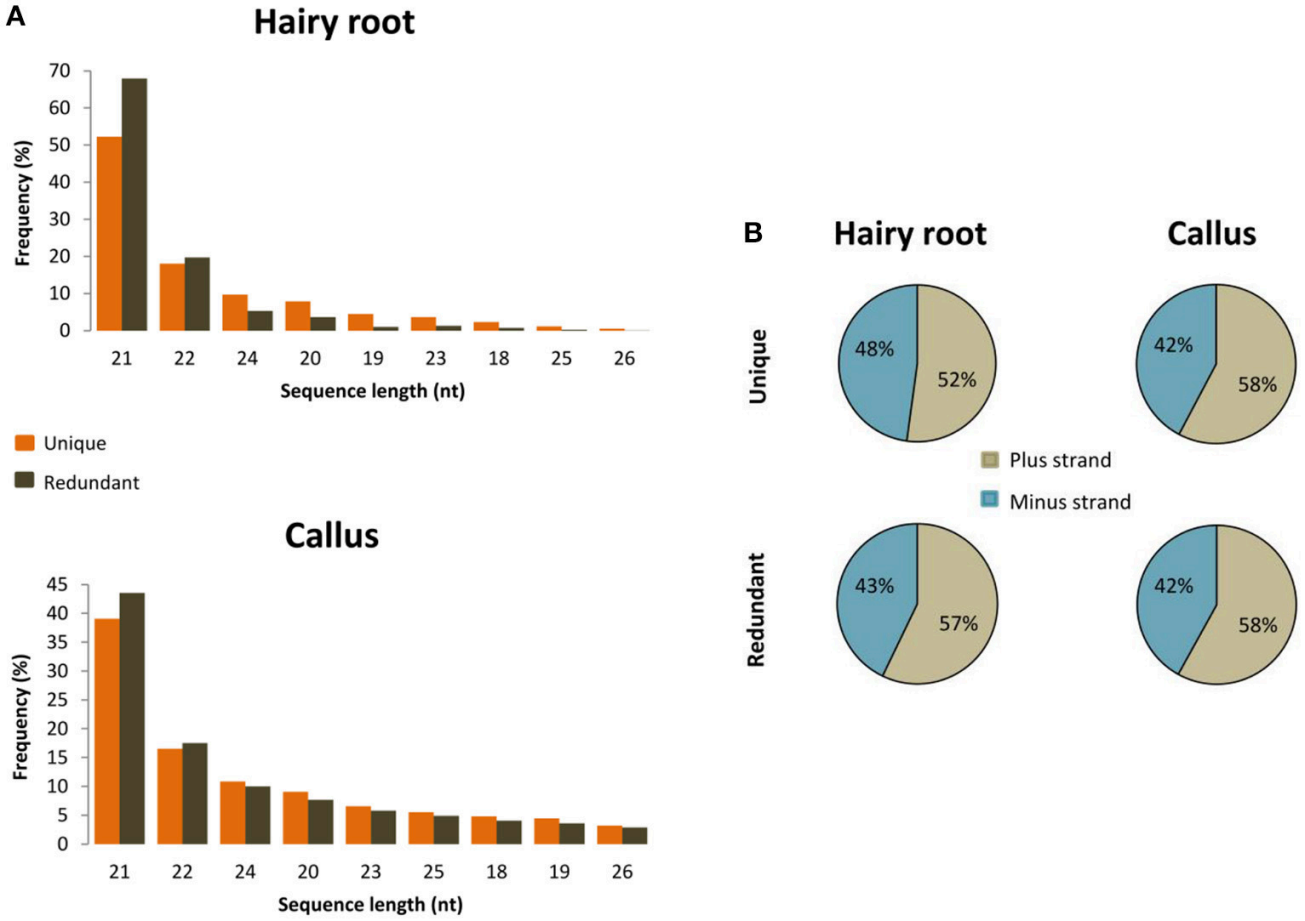
**Size and orientation of ArT-sRNAs. (A)** Sequence length distribution of ArT-sRNAs found in the hairy roots and *A. rhizogenes*-induced calli libraries that aligned to the T-DNA. **(B)** Average percentage of unique and redundant sequences that aligned with the plus strand (gray) and the minus strand (blue) of the T-DNA for each of the small RNA libraries.

**Figure 4:**
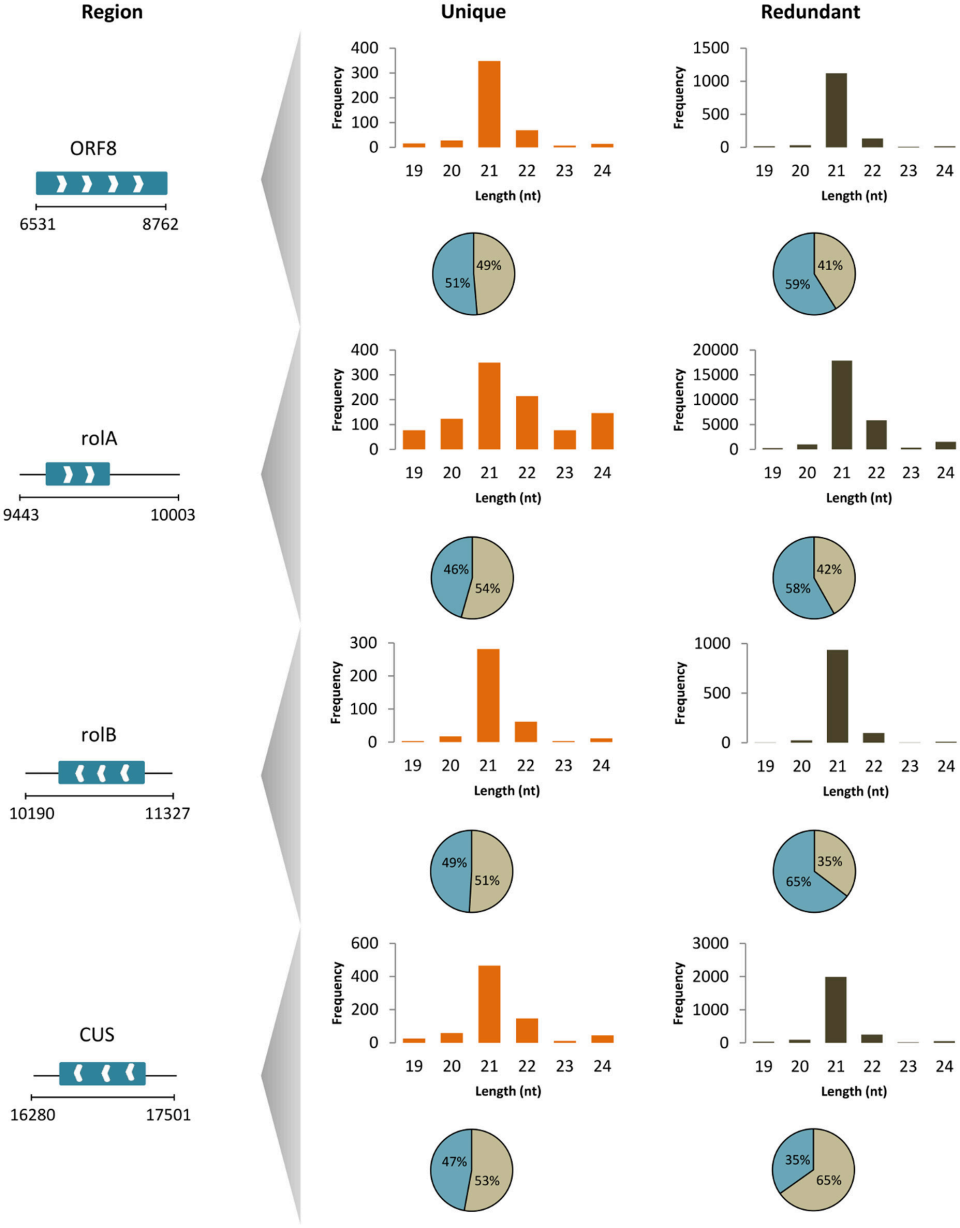
**ArT-sRNAs from hairy roots mapped to T-DNA genes**. Length distribution (column plots) and strand origin (circular plots; positive strand gray, negative strand blue) of unique and redundant reads of ArT-sRNAs for particular T-DNA genes (region according to base-pair positions in gb: EF433766). Genes encoded in the sense (white right arrows) and antisense (white left arrows) strand are shown.

**Figure 5:**
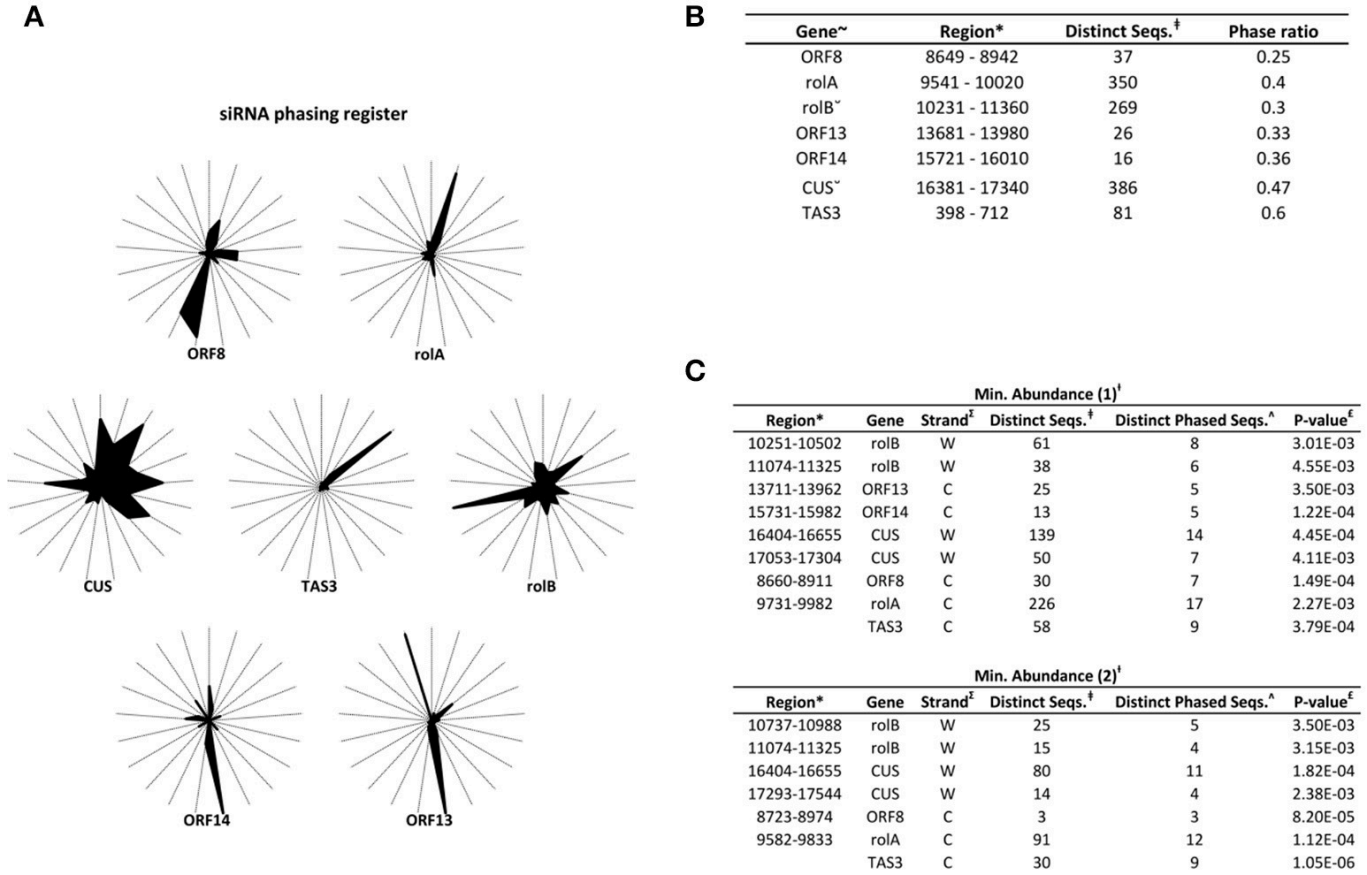
**PhasiRNA-producing loci from the T-DNA. (A)** Radial graphs representing the phasing register of ArT-sRNAs from hairy roots as performed by Axtell (2010). The 21 spokes of each graph correspond to the possible phasing registers. **(B)** Analysis of phasiRNA-producing loci represented in radial graphs. The genes (^~^), the region considered in the analysis (^*^) and the distinct small RNAs that mapped the region are indicated (‡). The region considered for the *rolB* and *CUS* genes is greater (ˇ). Loci with phase ratios of <0.25 were discarded. The *TAS3* transcript that is targeted by miR390 to generate ta-siRNAs was used as control. **(C)** Identification of phasiRNAs as described by Chen et al. (2007) taking into account ArT-sRNAs with one (upper part) or more than two (lower part) absolute reads (Ɨ). The strand of the loci (∑), the number of phased ArT-sRNAs (ʌ) and the *P*-value (£) are indicated.

To unravel the probable biogenesis and action of common bean ArT-sRNAs, their size, strand origin, and phased patterns were analyzed. It was found that ArT-sRNAs were largely small RNAs of 21-nt in length in hairy roots and calli (Figure 3A). In hairy roots, 52.2% of the ArT-sRNAs (unique sequences), representing 67.9% of the total sequences that aligned to the T-DNA, were of 21-nt in length. Only 5% of the total sequences corresponded to the 24-nt class that is involved in transcriptional regulation. The 22-nt class of ArT-sRNAs was the second most diverse (18%) and abundant (19.7%). A similar pattern of accumulation was observed for the ArT-sRNAs detected in calli, which indicates that silencing of T-DNA transcripts may occur at the post-transcriptional level. Also, ArT-sRNAs were produced from the sense and antisense strands, suggesting that ArT-sRNAs may be generated from perfect long double stranded RNAs (Figure 3B). In hairy roots, 57% of redundant sequences of the ArT-sRNAs found aligned to the sense strand while 43% aligned to the antisense strand. Taking a closer look to particular genes encoded in the sense or antisense strand, the proportion of ArT-sRNAs that aligned to either strand was variable (Figure 4). Furthermore, phased secondary 21-nt small interfering RNAs characteristic of PHAS loci were identified for *ORF8, rolA, rolB, ORF13, ORF14* and *CUS* genes (Figures 5A–C). Genes like *rolA, ORF8, ORF13*, and *ORF14* exhibited one sRNA cluster and one predominant phase, whereas *rolB* and *CUS* presented two clusters without one main phase. Some phased small RNAs of the *rolA* transcript were among the most abundant sRNAs detected (Figure 5A).

### ArT-sRNAs-mediated cleavage of T-DNA transcripts

**Figure 6:**
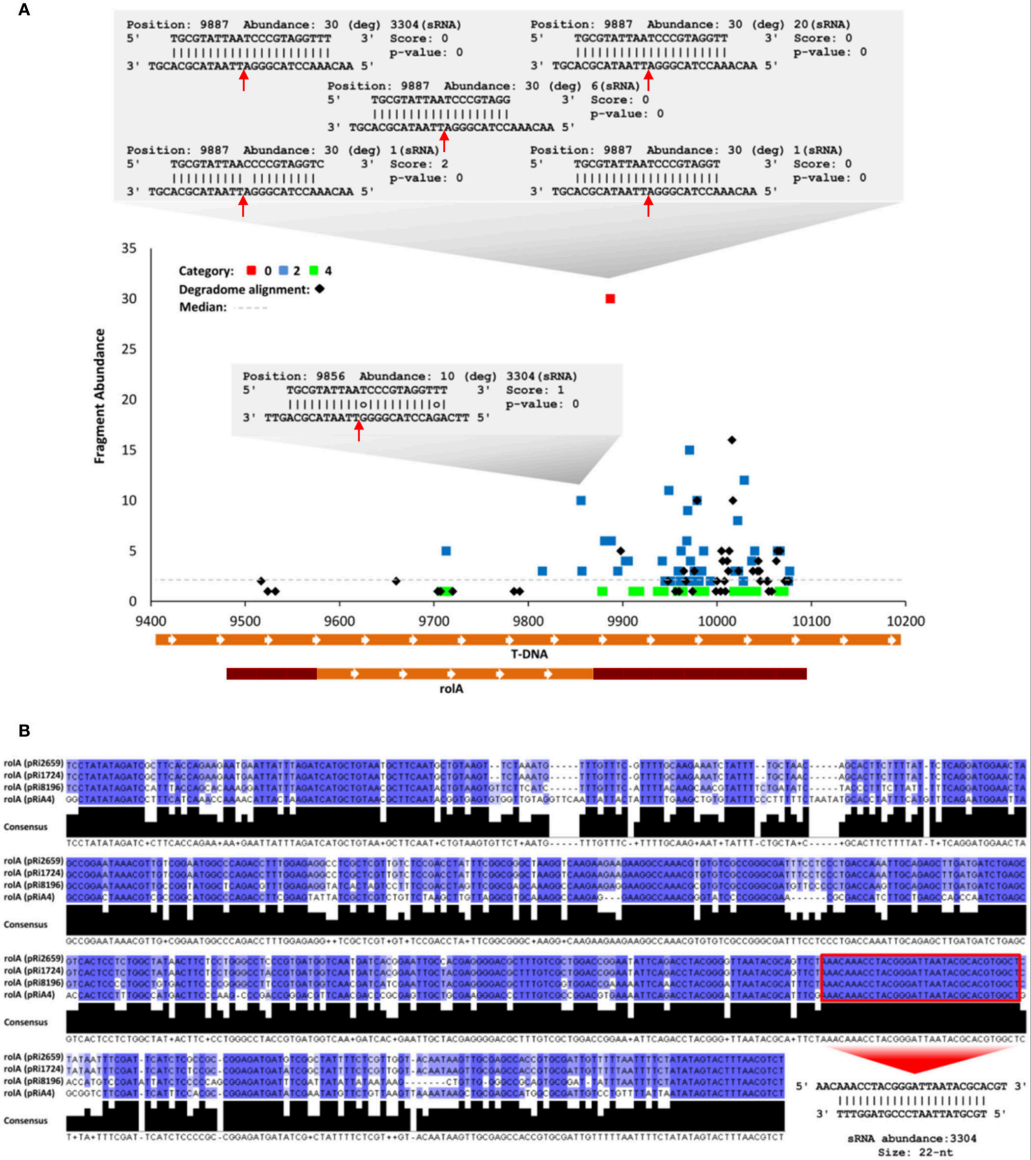
**Cleavage analysis of ***rolA*** by ArT-sRNAs. (A)** T-plot showing different categorized interactions (cleavage sites) between *rolA* and ArT-sRNAs. The interactions between *rolA* and the most abundant ArT-sRNA of 22-nt and its variants in length are shown (gray boxes). Cleavage sites for alignments are indicated (red arrows). In the T-DNA (X-axis), the coding region for *rolA* is indicated at the bottom (orange box) and the 5′ and 3′ untranslated regions (crimson red boxes). **(B)** Multiple alignment of *rolA* sequences from different Ri plasmids. The ArT-sRNA of 22-nt is indicated (box and red arrow).

Forty percent of the ArT-sRNAs of 21-nt in length that were detected in this analysis had a 5′ terminal uracil (Supplementary Figure 3). Small RNAs with this characteristic are recruited by AGO1. To gain insight into the cleavage of T-DNA transcripts through AGOs/ArT-sRNAs associations, a degradome (also called parallel analysis of RNA ends, PARE) library from hairy roots was deep-sequenced and analyzed. Sequence alignment resulted in the identification of 446 distinct sequences that aligned perfectly to the T-DNA of *A. rhizogenes*. Degradome sequences corresponding to all of the coding sequences of the T-DNA genes except for *ORF13* were detected (Supplementary Figures 4, 5). Most of the reads detected had the sequence of the *rolA* and *CUS* genes. Discovery analysis of sRNA/target interactions using degradome sequences and ArT-sRNAs from hairy roots exposed 180 interactions between ArT-sRNAs and the transcripts of the T-DNA with significant *p*-values at 73 unique cleavage positions (Supplementary Table 2). According to degradome read abundances (Addo-Quaye et al., 2009), 5 interactions fell into category 0 (highly likely to occur), 116 into category 2, and 59 into category 4. From almost all of the interactions found, around 95.5%, pointed out to cleavage sites of the *rolA* transcript, mainly from the 3′ untranslated region (Figure 6A). These results strongly suggest that AGOs/ArT-sRNAs associations occur. Furthermore, one of the ArT-sRNAs that fell into category 0, and thus cleaved the most abundant fragment in the transcript of *rolA*, was indeed the most abundant ArT-sRNA in the library. This ArT-sRNA of 22-nt in length with 3304 absolute read counts in the library presented another cleavage position including two mismatches (Figure 6A). This cleavage position in the transcript of *rolA* was consistent with the phasing register of the detected *rolA* phase (Figure 5A). In addition, the sequence of this ArT-sRNA was found to be highly conserved among *rolA* sequences of different Ri plasmids (pRi8196, pRi1724, pRiA4, and pRi2659), which suggests functional roles for this small RNA (Figure 6B).

### Potential host gene silencing through ArT-sRNAs

ArT-sRNAs may present the required complementarity base pairing with the transcripts of *P. vulgaris* to cleave them. To search for complementarity base pairing between ArT-sRNAs and host transcripts (*P. vulgaris*), a target prediction analysis was performed using the plant small RNA target analysis server psRNATarget (Dai and Zhao, 2011). The identification of 399 unique ArT-sRNAs/target interactions using a stringent cut-off threshold of the complementarity score (Supplementary Table 3) was determined. The interactions detected were composed by 299 distinct transcripts and 326 distinct ArT-sRNAs. To provide further evidence of ArT-sRNAs-mediated cleavage of host gene transcripts, the degradome library obtained from hairy roots was analyzed in pursuit of putative interactions. MicroRNAs (Supplementary Table 4) were used for analysis of conserved validated target-sRNA interactions in the degradome library as controls. Most of the microRNA-target interactions found corresponded to conserved validated target-microRNA interactions (Supplementary Table 5). In total, 402 interactions between host targets and ArT-sRNAs were detected using PAREsnip with default parameters (Supplementary Table 6). The total number of unique host transcripts predicted to be cleaved by 346 unique ArT-sRNAs was 228. No interactions were found between ArT-sRNAs and these 228 transcripts using the degradome sequencing of non-transformed common bean seedlings (Formey et al., 2015). Most of the ArT-sRNAs that define these interactions were of 21-nt in length (53.4%). Taking into account all the interactions, 58 fell into category 0, 50 into category 1, 155 into category 2, 3 into category 3, and 136 into category 4. The host transcript targets identified correspond to a diverse set of protein families (Supplementary Table 7). Gene Ontology (GO) enrichment analysis was performed for the 98 of the unique host targets (Supplementary Table 8). The two most enriched gene sets within the cellular component category were cellular (GO: 0044464, *p*-value = 1.91E-09, FDR = 5.97E-07) and intracellular components (GO: 0044424, *p*-value = 2.97E-07, FDR = 6.96-05). Metabolic (GO: 0008152, *p*-value = 8.45E-12, FDR = 7.58E-08) and cellular processes (GO: 0009987, *p*-value = 7.68E-11, FDR = 3.44E-07) were the most enriched gene sets observed from the biological process category. The response to stress category was also detected by the GO enrichment analysis (GO: 0006950, *p*-value = 2.49E-08, FDR = 4.31E-05) suggesting probable regulation of host transcripts by ArT-sRNAs as a counter-defensive strategy. Given that most of the interactions found by PAREsnip were validated interactions (Folkes et al., 2012) and that we found several interactions between ArT-sRNAs and *P. vulgaris* transcripts, our findings suggest that ArT-sRNAs could target common bean transcripts.

## Discussion

*A. rhizogenes* pathogenicity is characterized by the insertion of the T-DNA into the plants' nuclear genome. For this reason, it represents a powerful system to transform plants (Tzfira and Citovsky, 2006; Georgiev et al., 2012). Also, it is capable to transforming plants that are recalcitrant to *A. tumefaciens* infection (Taylor et al., 2006). Recent progress on the understanding of the induction and regeneration of hairy roots has increased its usage in the production of recombinant proteins, metabolic engineering, and phytoremediation (Georgiev et al., 2012). In contrast to *A. tumefaciens, A. rhizogenes* is frequently used with the root-inducing plasmid “armed,” carrying the T-DNA oncogenes (Taylor et al., 2006). For years, *A. rhizogenes* oncogenes have been considered modulators of neoplastic growth and differentiation (Britton et al., 2008). Recently, they have been also identified as triggers of secondary metabolism (Bulgakov et al., 2016). The biochemical and molecular characterization of the T-DNA genes from *A. rhizogenes* is not completely understood (Britton et al., 2008). In several studies, conflicting results regarding their functions have been reported, suggesting that new meristem emergence and subsequent differentiation of transformed plant cells may be conducted through complex molecular mechanisms (Britton et al., 2008). In recent years, small RNAs have emerged as important regulators of gene expression during different biological processes (Chapman and Carrington, 2007). Although, RNA silencing mechanism has been reported to regulate transgenes, viral nucleic acids and genes from the T-DNA of *A. tumefaciens*, its regulatory activity against the T-DNA of *A. rhizogenes* in hairy roots had not been analyzed (Blevins et al., 2006; Deleris et al., 2006; Dunoyer et al., 2006).

In this study, several abundant small RNAs generated from the T-DNA transcripts of *A. rhizogenes* in hairy roots were detected. Interestingly, miR319 was the most abundant miRNA family in the library of calli. MiR159 was the most abundant miRNA in roots, flowers and seedlings of *P. vulgaris* (Peláez et al., 2012). The miR319 family targets TCP (TEOSINTE BRANCHED-CYCLOIDEA/PROLIFERATING CELL FACTORS) genes involved in hormone and signaling pathways, proliferation, and differentiation (Schommer et al., 2012). Reduced activity of TCP genes caused by the increase of miR319 accumulation intensifies cellular proliferation (Schommer et al., 2012). Based on the findings above, this miRNA could be crucial for calli formation. MicroRNAs miR408 and miR394 were up-regulated in hairy roots and non-transgenic roots compared with calli. MiR408 targets genes of the phytocyanin family (cupredoxin, plnatacyanin, and uclacyanin) and a laccase (Abdel-Ghany and Pilon, 2008; Zhang and Li, 2013). MiR408 has been reported to play a role in abiotic stress responses and in vegetative development in *Arabidopsis* (Zhang and Li, 2013; Ma et al., 2015). *Arabidopsis* plants overexpressing miR408 presented enhanced root development under salinity stress and increased tolerance to oxidative stress (Ma et al., 2015). Oxidative and salinity stress tolerance have been reported for hairy roots (Bulgakov et al., 2013). Probably accumulation of miR408 contributes to these abiotic stress responses in hairy roots. MiR394 targets the gene LCR (LEAF CURLING RESPONSIVENESS), an F-box protein (SKP1-Cullin/CDC53-F-box). Roles described by Knauer et al. (2013) for miR394 makes it a highly attractive microRNA to study in *Agrobacterium*-plant interactions. MiR394 was found to be essential for shoot meristem formation. Interestingly, stem cells differentiate in the miR394 mutant plants (Knauer et al., 2013). In this study, miR394 was poorly detected in calli, suggesting a role for this microRNA in hairy root emergence. MiR393, a microRNA well known to be involved in plant defense responses to bacterial infection, was upregulated in calli when compared with hairy roots. MiR393 was almost undetectable in both roots (Figure 2A). Induced accumulation of miR393 was observed during *A. tumefaciens* infiltration at an early stage of infection (Pruss et al., 2008). MiR393 targets three auxin receptors: TIR1, AFB2, and AFB3. Besides its roles in plant defense responses, we consider that miR393 may play an important role during the hairy root disease because of auxin relevance in calli formation and organ induction (Ikeuchi et al., 2013).

Among the T-DNA transcripts producing ArT-sRNAs, the *rolA* transcript stands out as a major source of small RNAs. The precise function of the rolA protein is still unknown (Britton et al., 2008). Poor expression of *rolA* has been reported in roots and in leaves (Carneiro and Vilaine, 1993). Tobacco plants expressing *rolA* present wrinkled leaves, shortened internodes, and abnormal flowers (Carneiro and Vilaine, 1993). Local expression of *rolA* in the vascular bundles reduces the size of the surrounding parenchyma cells causing the wrinkled leaf phenotype. For this reason, and based on reciprocal grafting studies involving *rolA*, it has been proposed that *rolA* generates a diffusible factor (Guivarc'h et al., 1996; Britton et al., 2008). Given their nature, ArT-sRNAs derived from *rolA* may constitute this diffusible factor. ArT-sRNAs could also indirectly impact on plant phenotype through altering endogenous sRNA-regulated processes as described during PTGS of foreign genetic material (Martínez de Alba et al., 2011). Intriguingly, no ArT-sRNAs were derived from the *rolC* gene in hairy roots, one of the T-DNA genes widely studied as well. Strong and stable expression of *rolC* in roots of transformed plants previously reported together with the null detection of ArT-sRNAs generated from this gene, suggests that *rolC* is poorly or not at all subjected to silencing in hairy roots (Sugaya et al., 1989).

The detection of ArT-sRNAs, mainly of 21- and 22-nt in length, suggests that the T-DNA genes may be silenced at the post-transcriptional level and that their transcripts are probably processed by DCL4 and DCL2 as occurs during regulation of RNA virus (Deleris et al., 2006). Also, the identification of ArT-sRNAs as phasiRNAs indicates an active role of one or more RDRs during the regulation of these genes. DCL4 often produces phasiRNAs of 21-nt from RDR6-dependent dsRNA that can be loaded into AGO1 (Fei et al., 2013). Besides, cleavage analysis of the T-DNA transcripts by ArT-sRNAs indirectly shows that this small RNAs could be loaded into AGO protein-complexes. Most of them could be loaded onto AGO1, which has an important role in antiviral silencing (Peláez and Sanchez, 2013). The interactions detected for conserved microRNAs using the PAREsnip algorithm and the degradome data were mainly validated interactions. For this reason, the amount of interactions detected between ArT-sRNAs and host transcripts suggests that ArT-sRNAs may help shape hairy root phenotype through direct regulation of the host transcripts. Further experiments are required to truly validate this regulation. Although, its known that virus-derived small interfering RNAs and fungal small RNAs regulate host genes like endogenous sRNAs (Qi et al., 2009; Shimura et al., 2011; Smith et al., 2011; Weiberg et al., 2013).

RNA silencing of transcripts from the T-DNA of *A. rhizogenes* still has to be determined in hairy roots and calli because several factors could influence their accumulation levels in these two different samples. Transcripts from the T-DNA in hairy roots were detected by quantitative PCR (Supplementary Figure 1) but, the degree of silencing for these transcripts still has to be evaluated. Also, more experiments have to be done to define if poor accumulation of ArT-sRNAs and reduced accumulation of most miRNAs in calli are a consequence of the amount of transformed cells or a RNA silencing suppression state similar to *A. tumefaciens*-induced tumors. It is also possible that ArT-sRNAs detected in calli belong to cells that have begun the process of differentiation. Nevertheless, these results contribute to the understanding and usage of hairy roots as an experimental model. We propose that, if the T-DNA genes are silenced in the hairy root disease, one may consider the population of ArT-sRNAs when designing constructs for its overexpression in hairy roots to avoid possible silencing. Also, it would be interesting to determine if the role of RNA silencing against the T-DNA genes has an impact on the efficiency of constructs designed to silence genes. For this reason, studies have to take into account these observations when elucidating the function of several T-DNA genes, specially of *rolA*. Our results also suggest that additional genes of the T-DNA of *A. tumefaciens* could be silenced at the beginning of the infection (Dunoyer et al., 2006). Future studies may reveal if regulation of the T-DNA transcripts by RNA silencing contributes to hairy root emergence or phenotype.

## Author contributions

PP conceived the study. PP wrote the manuscript and carried out plant growth, RNA extraction, and preparation and the bioinformatic analyses. PP and AH carried out the quantitative PCR analysis. GE and FS provided intellectual suggestions. Authors read and approved the final manuscript.

## Funding

Financial support for this research was provided by the Consejo Nacional de Ciencia y Tecnología (CONACYT) and Programa de Apoyo a Proyectos de Investigación e Innovación Tecnológica (PAPIIT-DGAPA-IN206415). PP was supported by a fellowship from CONACYT.

### Conflict of interest statement

The authors declare that the research was conducted in the absence of any commercial or financial relationships that could be construed as a potential conflict of interest.
