# Supplementary material for: Small RNAs Derived from the T-DNA of Agrobacterium rhizogenes in Hairy Roots of Phaseolus vulgaris
Source: Front Plant Sci. 2017 Feb 1;8:96. doi: 10.3389/fpls.2017.00096 (PMC5285386; doi:10.3389/fpls.2017.00096)
Supplement: Supplementary file 12 [file DataSheet1.DOCX]

Small RNAs Derived from the T-DNA of Agrobacterium rhizogenes in Hairy Roots of Phaseolus vulgaris

Pablo Peláez^*^, Alejandrina Hernández-López, Georgina Estrada-Navarrete, Federico Sanchez.

*** Correspondence:** Pablo Peláez: [pablo.pelaez@cinvestav.mx](mailto:pablo.pelaez@cinvestav.mx)

# Supplementary Figures and Tables legends

**Supplementary Figure 1.** Quantitative PCR analysis of T-DNA transcripts in hairy roots. The expression of transcripts was normalized with the reference gene *EF1*. Error bars represent standard deviation (n=3).

**Supplementary Table 1.** Small RNAs (18-26nt) derived from the Ri plasmid T-DNA region**.** Abundance and sequence of ArT-sRNAs found in the small RNA libraries of callus and hairy roots.

**Supplementary Figure 2.** Differential accumulation analysis of microRNA families miR319, miR394 and miR408 from hairy roots, calli and non-transgenic roots libraries. Frequencies of reads were normalized (RPM) and represented in logarithmic scale (Y-axis).

**Supplementary Figure 3.** 5´ terminal nucleotide of T-DNA-derived sRNAs. Number of unique and redundant ArT-sRNAs (20-24 nt) from hairy roots and callus libraries with a particular 5´ terminal nucleotide. Red numbers represent the sum total of raw reads from a column or row.

**Supplementary Figure 4.** Alignment of degradome sequences to the T-DNA. Abundance and distribution of degradome sequences derived from the T-DNA. The sequences that aligned to the plus strand (yellow bars) and to the minus strand (green bars) of the T-DNA of *A. rhizogenes* (blue long bar) from hairy roots. In the bottom, the coding regions and the borders from the T-DNA are represented (blue bars).

**Supplementary Figure 5.** Number and location of raw degradome reads derived from the T-DNA. Unique and redundant raw degradome reads produced from genes of the T-DNA (region according to base-pair positions in gb: EF433766). The number of raw reads of a particular location from the plus or minus strand are indicated (Ɨ).

**Supplementary Table 2.** PAREsnip analysis of the T-DNA derived sRNAs against the degradome fragments aligning the T-DNA. Table of results generated from PAREsnip analysis.

**Supplementary Table 3.** Predicted targets for T-DNA derived sRNAs in *P. vulgaris.* Table of results of the plant small RNA target analysis server psRNATarget. Only ArT-sRNAs found in hairy roots were used.

**Supplementary Table 4.** MicroRNAs used for the identification of validated targets. List of microRNAs used as input in the PAREsnip analysis together with common bean primary transcripts (Phytozome v9.0).

**Supplementary Table 5.** Analysis of conserved miRNA targets. Table of results generated from PAREsnip analysis. Name and description of the common bean miRNA targets were included.

**Supplementary Table 6.** PAREsnip analysis of the T-DNA derived sRNAs against the transcripts of *P. vulgaris*. Table of results generated from PAREsnip analysis. ArT-sRNAs found in hairy roots and common bean primary transcripts were used as input.

**Supplementary Table 7.** Identification of *P. vulgaris* targets from PAREsnip analysis. Common bean homologs in *Arabidopsis* were used for annotation of targets through GoMapMan.

**Supplementary Table 8.** Gene ontology enrichment analysis of host transcript targets. GO categorization of 98 predicted common bean targets of ArT-sRNAs using the plant geneset enrichment analysis toolkit PlantGSEA (FDR < 0.05; background : *A. thaliana*).

**Supplementary Table 9.** Phasing-generating loci of *Phaseolus vulgaris*. Identification of phasing-generating loci of common bean as described by Chen et al. (2007). Small RNAs with one (upper part) or more than two (lower part) absolute reads (Ɨ) were considered. The transcript ID(~), the phase region (*), the strand of the loci (Ʃ), the number of phased small RNAs (ʌ) and the P-value are indicated (£).

**Supplementary Table 10.** Oligonucleotide probes used for miRNA detection by northern blot. Oligonucleotide probes for miR167a (21-nt) and for miR167d (22-nt) were included.

**Supplementary Table 11.** Primers used for qRT-PCR.

# Supplementary Figures


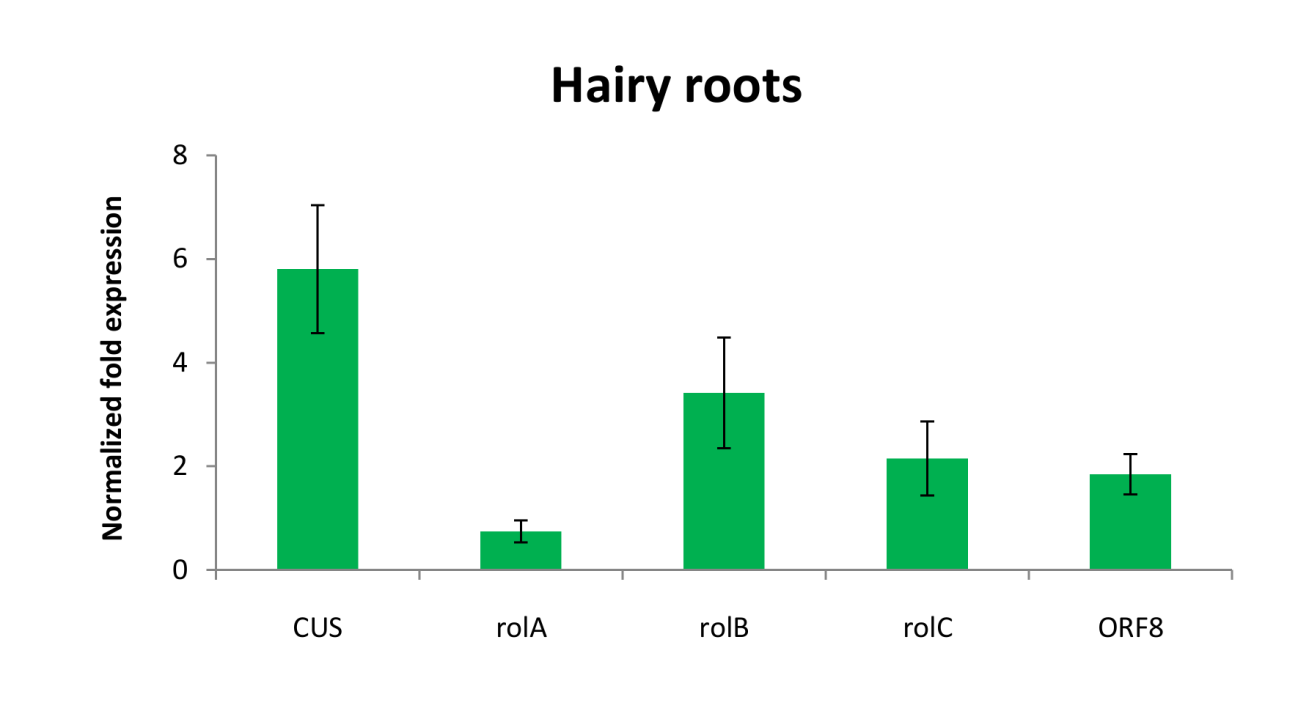


**Supplementary Figure 1.** Quantitative PCR analysis of T-DNA transcripts in hairy roots. The expression of transcripts was normalized with the reference gene *EF1*. Error bars represent standard deviation (n=3).


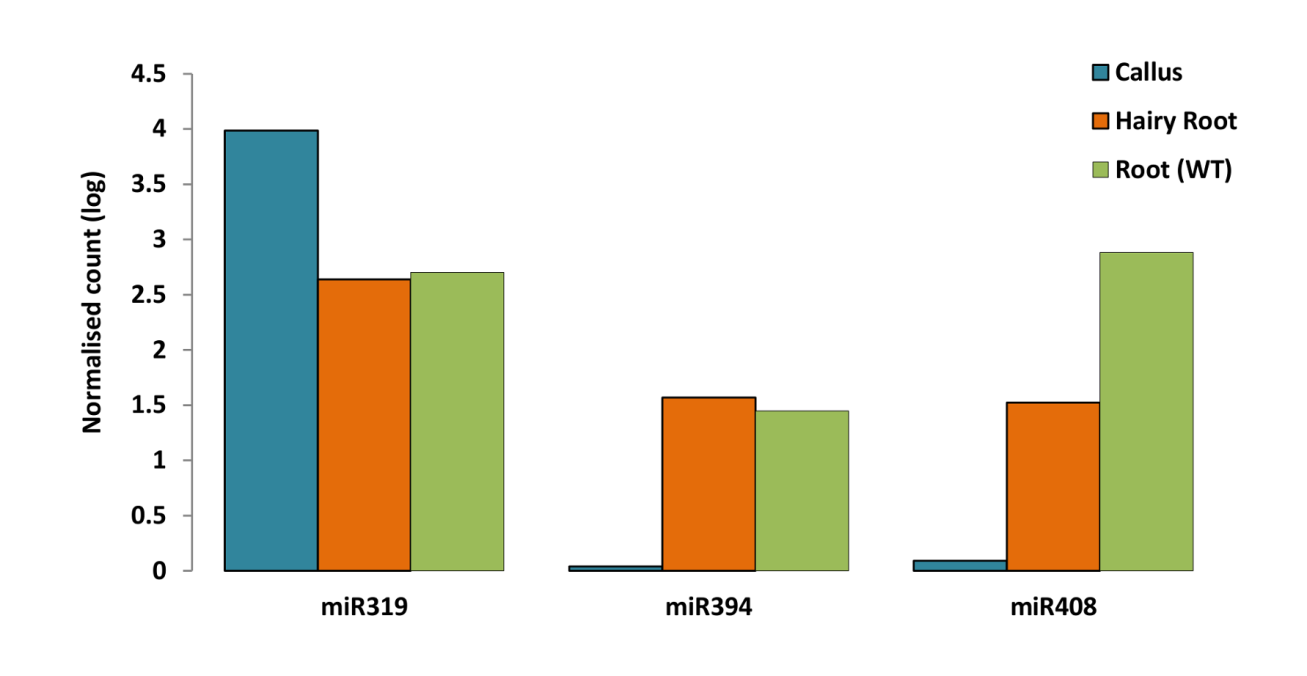


**Supplementary Figure 2.** Differential accumulation analysis of microRNA families miR319, miR394 and miR408 from hairy roots, calli and non-transgenic roots libraries. Frequencies of reads were normalized (RPM) and represented in logarithmic scale (Y-axis).


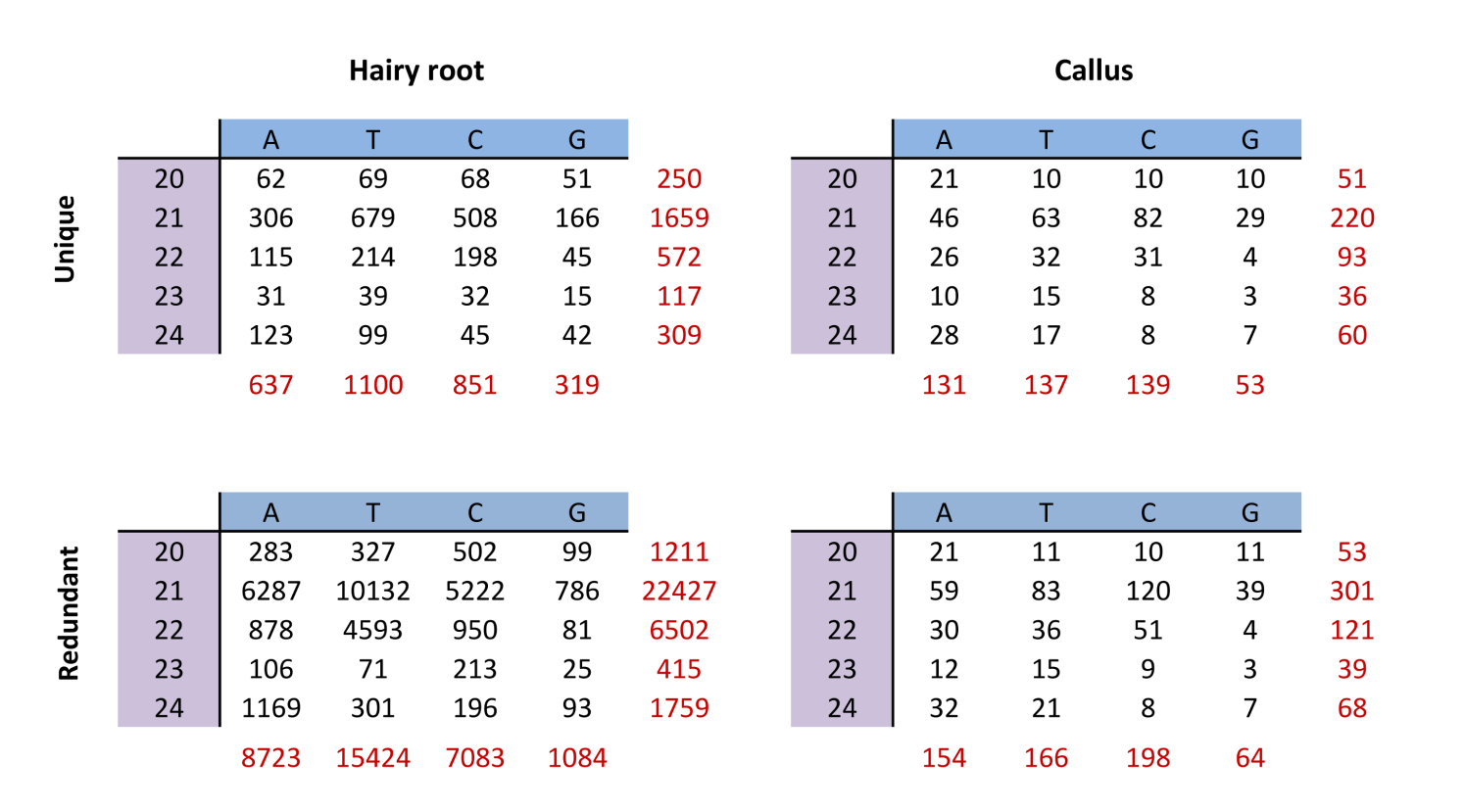


**Supplementary Figure 3.** 5´ terminal nucleotide of T-DNA-derived sRNAs. Number of unique and redundant ArT-sRNAs (20-24 nt) from hairy roots and callus libraries with a particular 5´ terminal nucleotide. Red numbers represent the sum total of raw reads from a column or row.


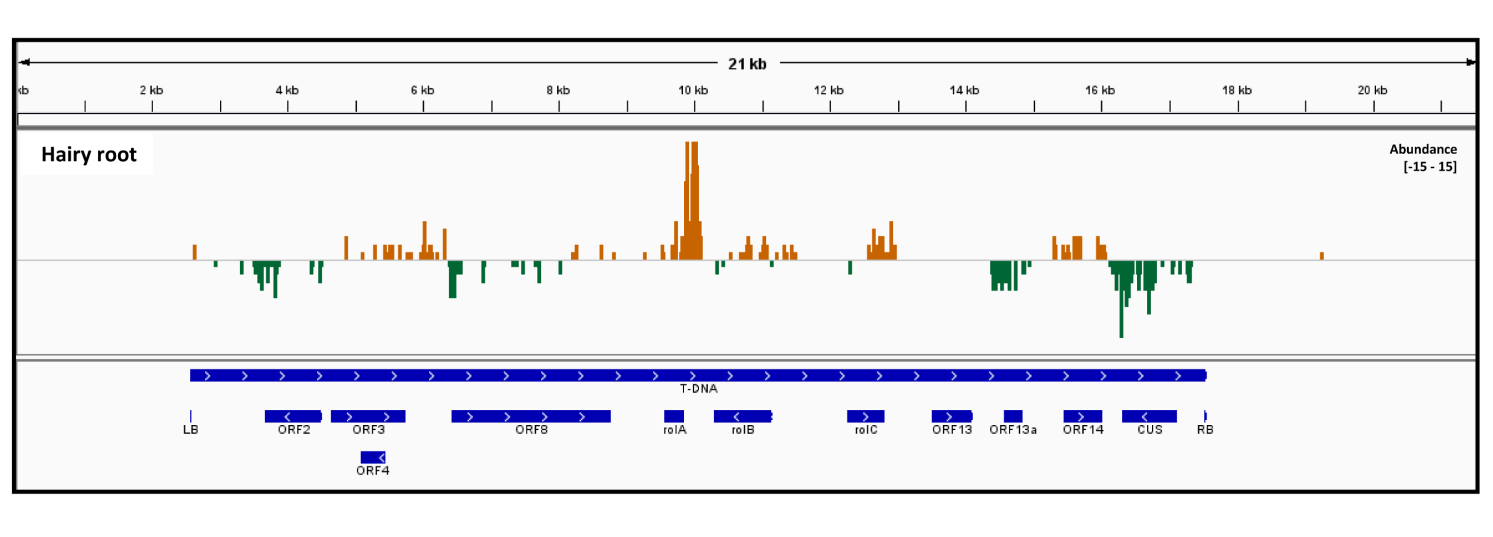


**Supplementary Figure 4.** Alignment of degradome sequences to the T-DNA. Abundance and distribution of degradome sequences derived from the T-DNA. The sequences that aligned to the plus strand (yellow bars) and to the minus strand (green bars) of the T-DNA of *A. rhizogenes* (blue long bar) from hairy roots. In the bottom, the coding regions and the borders from the T-DNA are represented (blue bars).


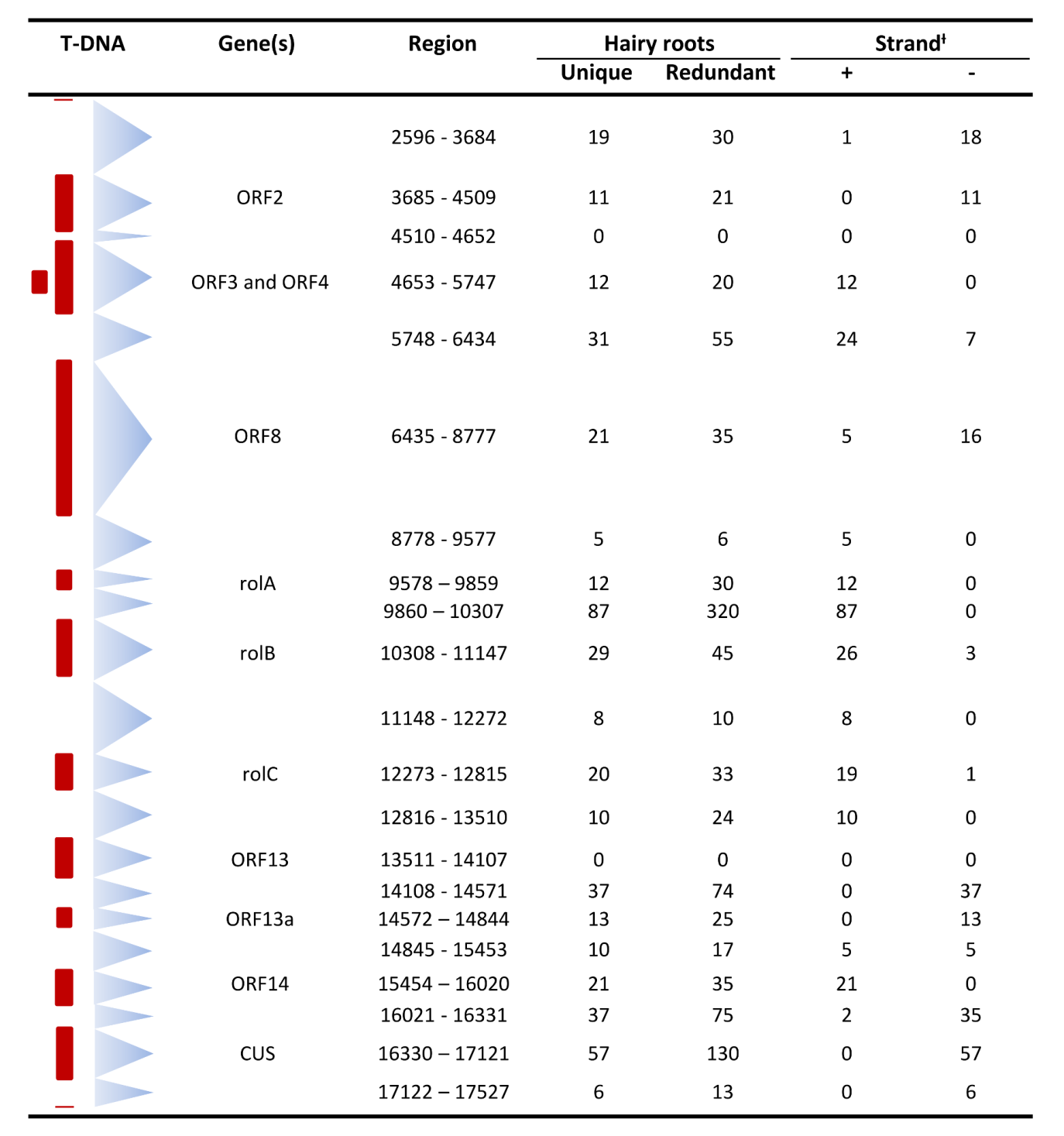


**Supplementary Figure 5.** Number and location of raw degradome reads derived from the T-DNA. Unique and redundant raw degradome reads produced from genes of the T-DNA (region according to base-pair positions in gb: EF433766). The number of raw reads of a particular location from the plus or minus strand are indicated (Ɨ).
