# Supplementary figures and images for: Small RNAs Derived from the T-DNA of Agrobacterium rhizogenes in Hairy Roots of Phaseolus vulgaris
Source: Front Plant Sci. 2017 Feb 1;8:96. doi: 10.3389/fpls.2017.00096 (PMC5285386; doi:10.3389/fpls.2017.00096)

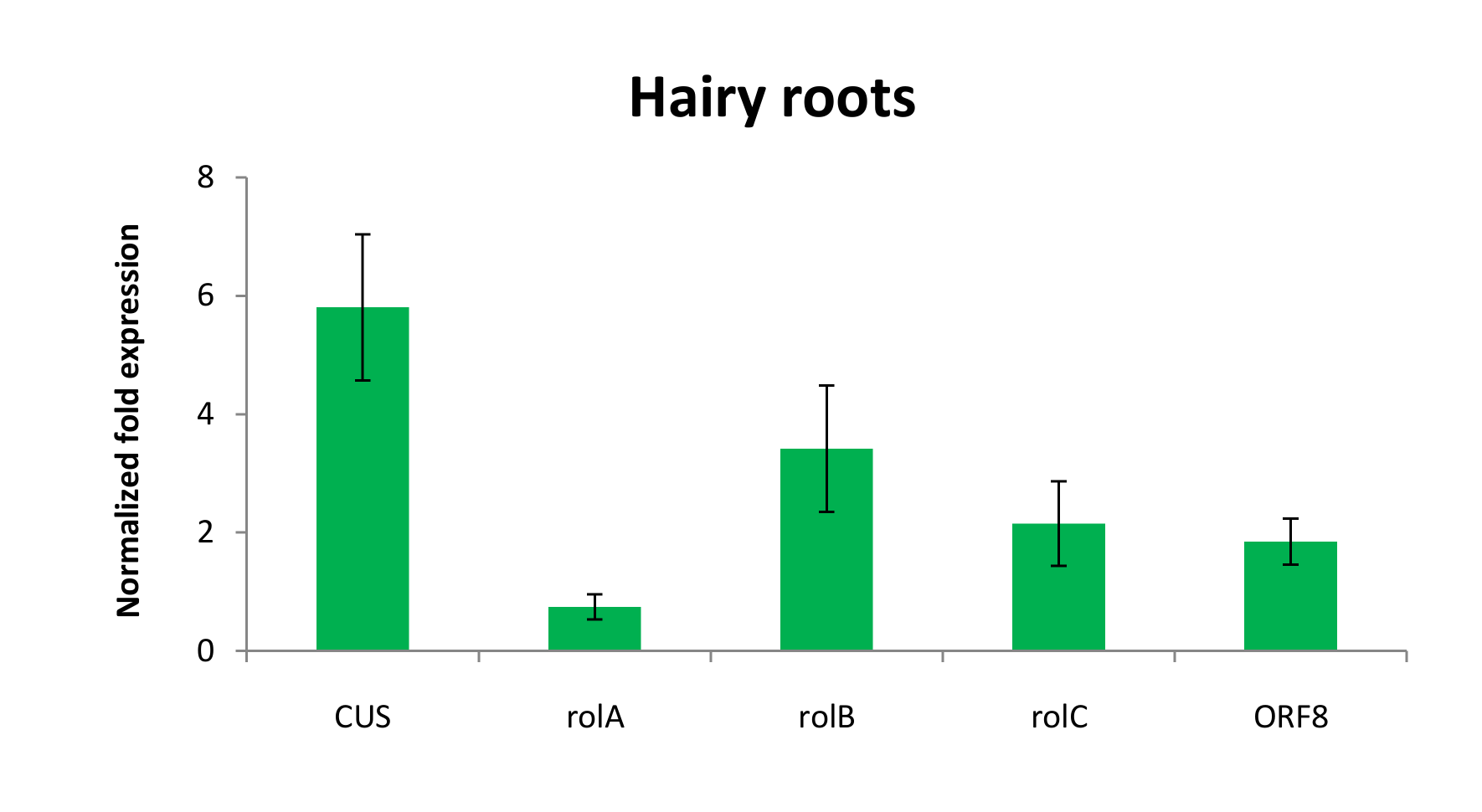

Supplement: Supplementary file 13 [file Image1.TIF]

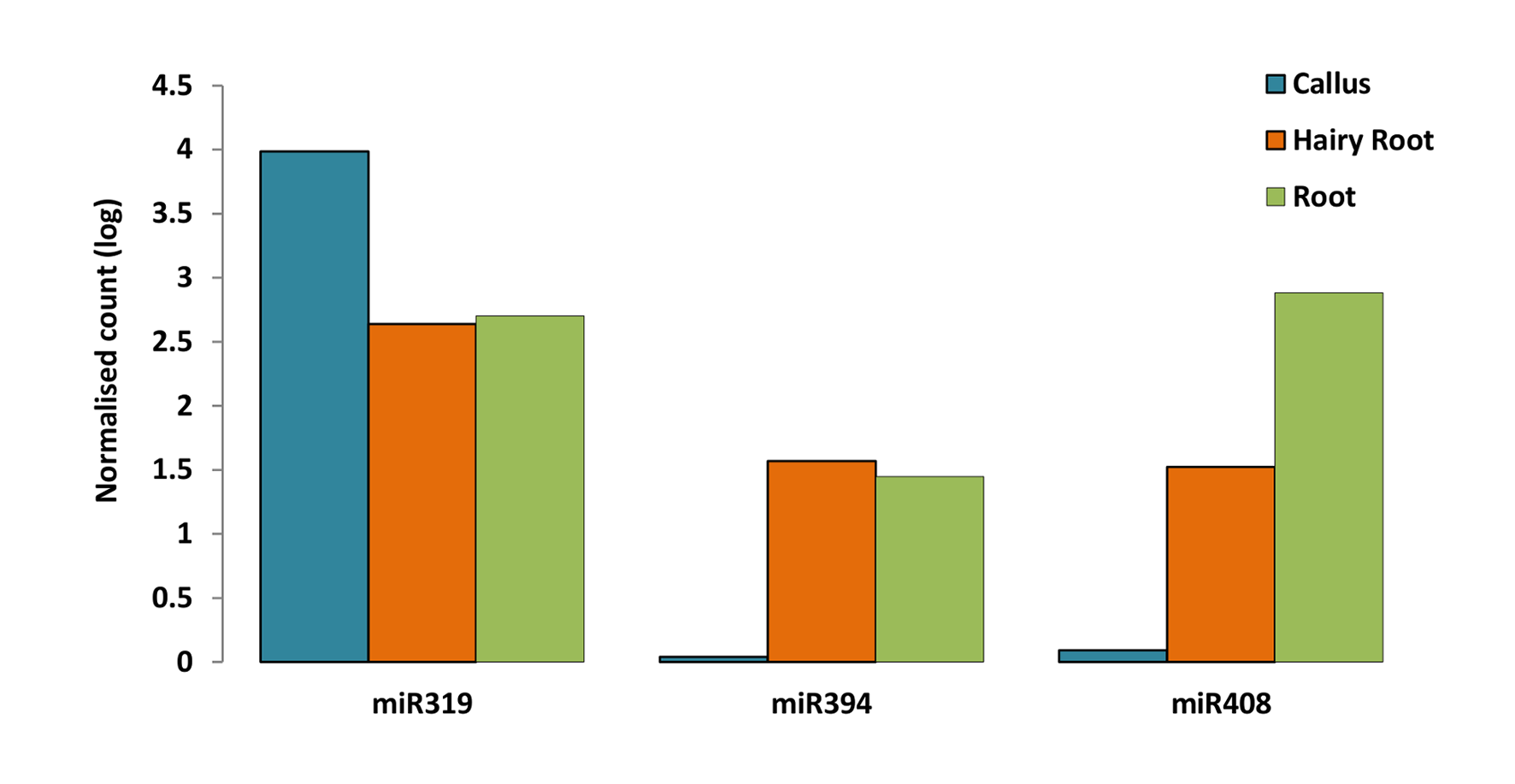

Supplement: Supplementary file 14 [file Image2.TIF]

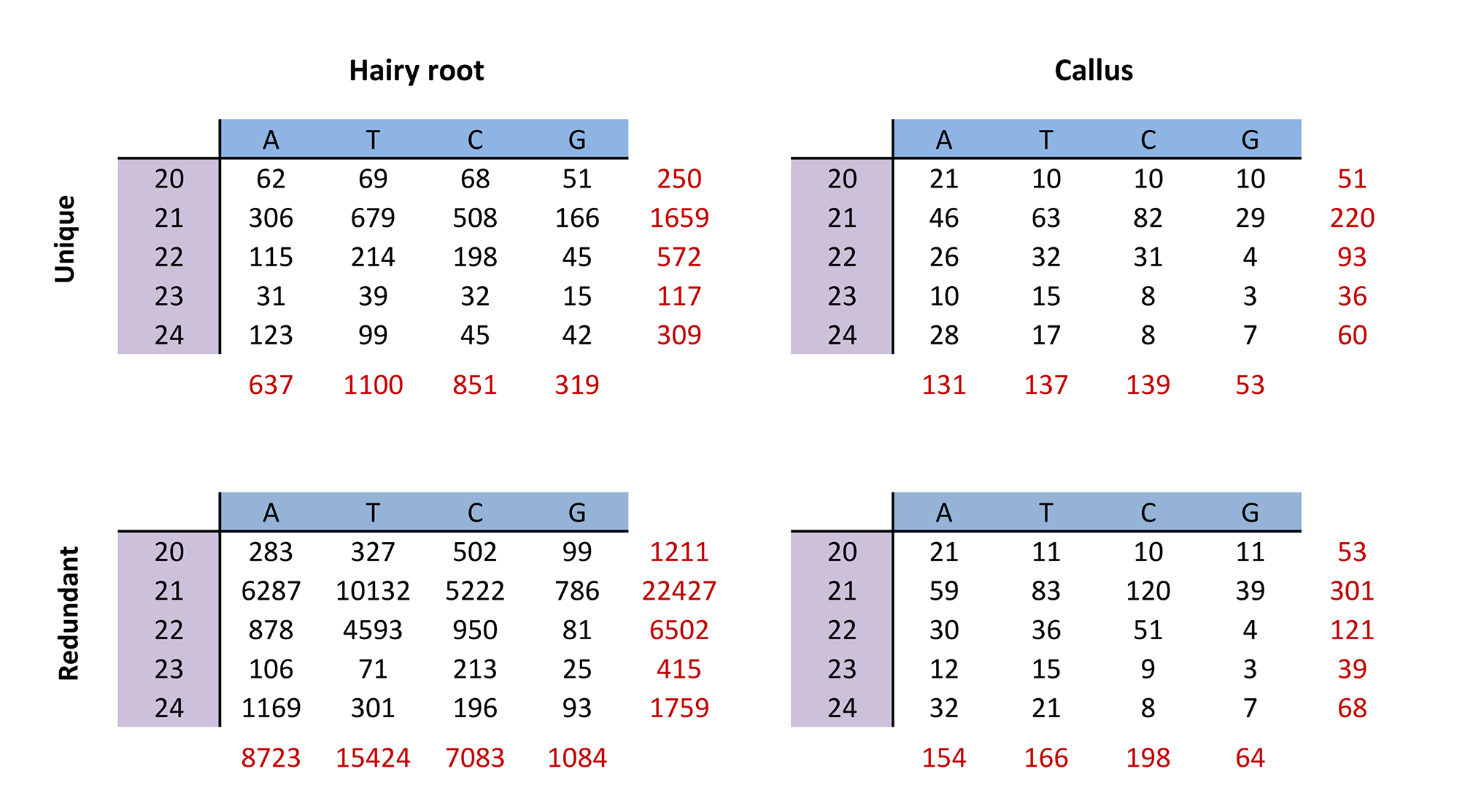

Supplement: Supplementary file 15 [file Image3.TIF]

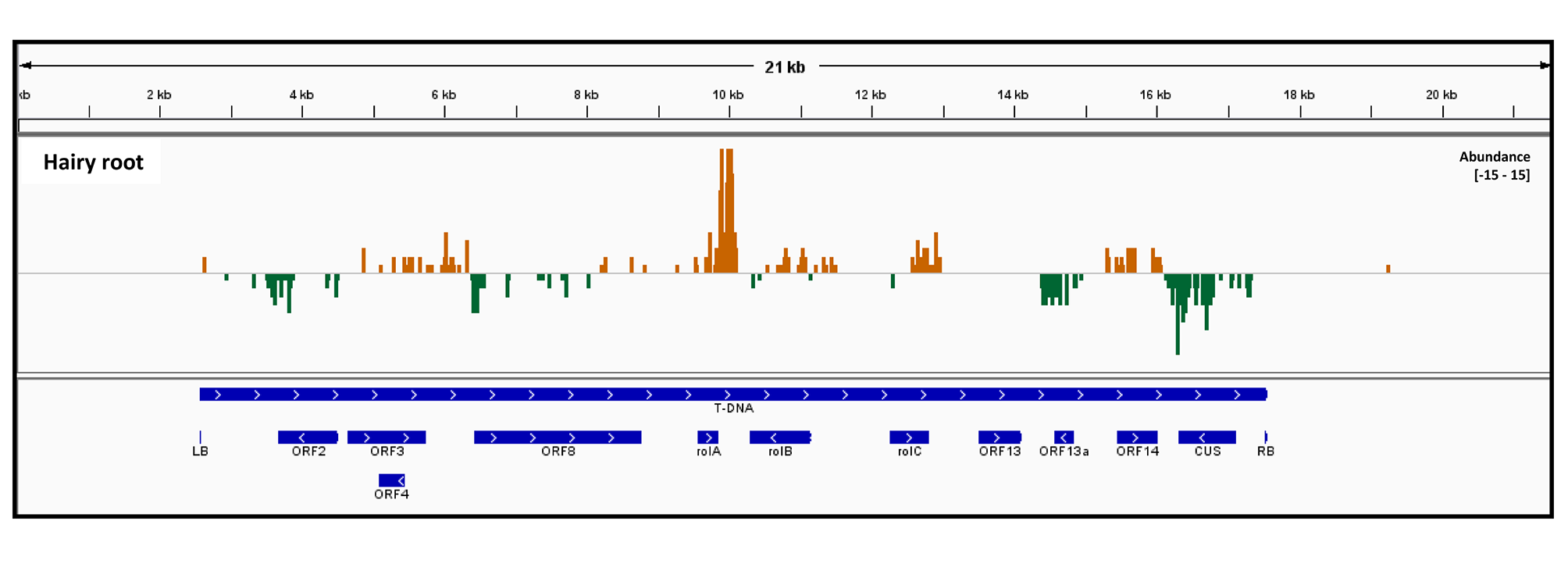

Supplement: Supplementary file 16 [file Image4.TIF]

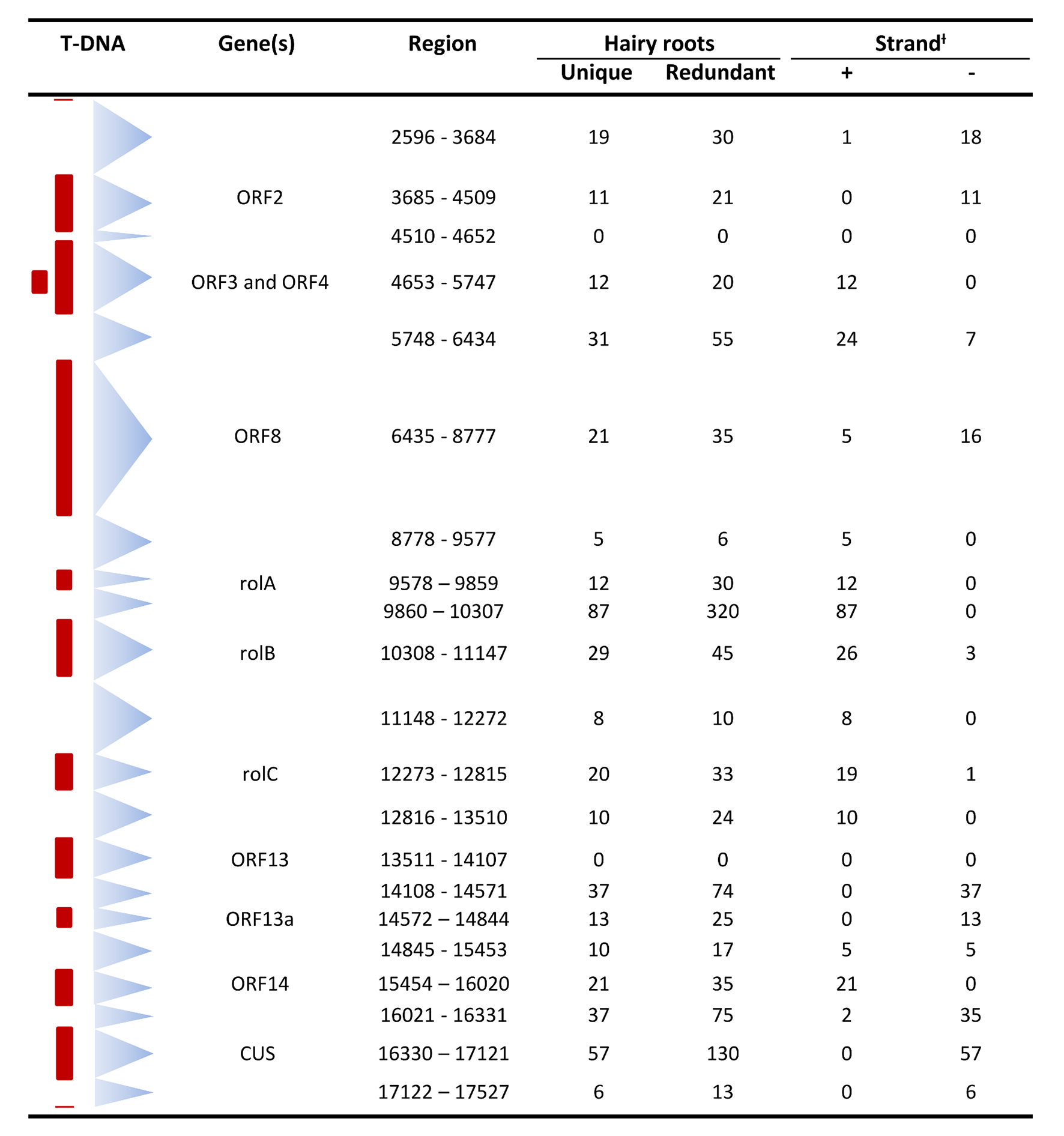

Supplement: Supplementary file 17 [file Image5.TIF]
